# Eukaryotic Cell Permeabilisation to Identify New Putative Chlamydial Type III Secretion System Effectors Secreted within Host Cell Cytoplasm

**DOI:** 10.3390/microorganisms8030361

**Published:** 2020-03-03

**Authors:** Carole Kebbi-Beghdadi, Ludovic Pilloux, Virginie Martin, Gilbert Greub

**Affiliations:** Center for Research on Intracellular Bacteria, Institute of Microbiology, Centre Hospitalier Universitaire Vaudois, CH-1011 Lausanne, Switzerland

**Keywords:** *Chlamydia trachomatis*, *Waddlia chondrophila*, Type III Secretion System, effectors, perfringolysin O, selective permeabilisation, host-pathogen interactions, virulence factors, nuclear effector

## Abstract

*Chlamydia trachomatis* and *Waddlia chondrophila* are strict intracellular bacteria belonging to the *Chlamydiales* order*. C. trachomatis* is the most frequent bacterial cause of genital and ocular infections whereas *W. chondrophila* is an opportunistic pathogen associated with adverse pregnancy outcomes and respiratory infections. Being strictly intracellular, these bacteria are engaged in a complex interplay with their hosts to modulate their environment and create optimal conditions for completing their life cycle. For this purpose, they possess several secretion pathways and, in particular, a Type III Secretion System (T3SS) devoted to the delivery of effector proteins in the host cell cytosol. Identifying these effectors is a crucial step in understanding the molecular basis of bacterial pathogenesis. Following incubation of infected cells with perfringolysin O, a pore-forming toxin that binds cholesterol present in plasma membranes, we analysed by mass spectrometry the protein content of the host cell cytoplasm. We identified 13 putative effectors secreted by *C. trachomatis* and 19 secreted by *W. chondrophila*. Using *Y. enterocolitica* as a heterologous expression and secretion system, we confirmed that four of these identified proteins are secreted by the T3SS. Two *W. chondrophila* T3SS effectors (hypothetical proteins Wcw_0499 and Wcw_1706) were further characterised and demonstrated to be early/mid-cycle effectors. In addition, Wcw_1706 is associated with a tetratricopeptide domain-containing protein homologous to *C. trachomatis* class II chaperone. Furthermore, we identified a novel *C. trachomatis* effector, CT460 that localises in the eukaryotic nucleus when ectopically expressed in 293 T cells.

## 1. Introduction

*Chlamydia trachomatis* is an important human pathogen responsible for genital tract or ocular infections that affect millions of people worldwide and can lead to infertility or blindness, depending on the different serovars [1,2]. *Waddlia chondrophila* is an emerging human and animal bacterial pathogen. In humans, its association with adverse pregnancy outcomes was documented on multiple occasions in the past ten years [3,4,5,6,7,8] and its implication in respiratory infections was also suggested [9,10]. *W. chondrophila* was isolated twice from an aborted bovine foetus and since then was reported to be a bovine abortigenic agent in several studies [10,11,12,13,14,15]. Consistent with its pathogenic potential, *W. chondrophila* is able to infect and propagate in human macrophages, the first line of defence against infection, as well as in endometrial cells and pneumocytes [16,17].

*C. trachomatis* and *W. chondrophila* both belong to the *Chlamydiales* order, a phylum that encompasses strict intracellular bacteria that are important human and animal pathogens as well as environmental or symbiotic organisms [18]. They share with all other members of the phylum a biphasic developmental cycle that involves two morphologically distinct forms, an extracellular infectious Elementary Body (EB) and an intracellular replicative Reticulate Body (RB). Inside their host cell, bacteria reside and multiply in a vacuole called inclusion (reviewed in [19]).

Like other intracellular bacteria, *Chlamydiales* need to communicate and interact closely with their host cells in order to promote bacterial survival and establish a favourable environment to support their replication. For this purpose, they secrete proteins, called effectors, into the inclusion lumen and within their host cell cytosol. Chlamydial genomes encode several essential protein secretion systems such as the Sec system, which delivers proteins in the periplasmic space and can be coupled to Type II or Type V Secretion Systems for translocation in the lumen of the inclusion. *Chlamydiales* also possess a Type III Secretion System (T3SS) that spans the inclusion membrane and allows direct secretion into the host cell cytosol [20]. Structural components of the T3SS apparatus and chaperones required for maintenance of the effectors in a secretion-competent state are very well conserved between all known members of the *Chlamydiales* order [18]. Effector proteins however are largely species-specific and likely contribute towards the differences in the host specificity and intracellular trafficking observed between the various members of this phylum [20,21]. Indeed, while *C. trachomatis* replication is mainly restricted to mammalian cells [22,23], *W. chondrophila* is able to multiply in a broad range of hosts including protists, insect, fish and mammalian cell lines [17,24,25]. Furthermore, these two bacterial species display different trafficking after internalisation within their host cell. *W. chondrophila* recruits mitochondria around the replicative vacuole whereas *C. trachomatis* disrupts the host cell Golgi and obtains lipids by intercepting vesicular traffic to the plasma membrane [16,26,27]. Both of them are able to escape endocytic pathway and exhibit a close contact between endoplasmic reticulum and the inclusion membrane [16,28]. 

Chlamydial T3SS effectors are divided in two subsets: (i) soluble effectors secreted within host cytosol, and (ii) effectors translocated and inserted in the inclusion membrane, also called Incs (reviewed in [20]). The latter share a specific feature, a hydrophobic bilobed domain that inserts the protein within the inclusion membrane, leaving the amino and carboxyl termini accessible on the host cell cytosol side (reviewed in [19]). T3SS effectors display very poor sequence homologies, making them difficult to identify, and even if *C. trachomatis* Incs have been widely studied, virtually nothing is known about Incs secreted by *W. chondrophila*, as well as about soluble effectors secreted by both bacteria. Effector secretion is an active process with an effective cost for the bacteria. This implies that every secreted protein has an important function and plays an essential role in bacterial entry, survival or replication. Identifying those secreted effectors is a crucial step in understanding the molecular bases of chlamydial pathogenesis. 

In the present work, we use a selective permeabilisation method to create pores in the eukaryotic plasma membrane of infected cells without disrupting the inclusion membrane. We submit the released cytosolic content of infected cells to mass spectrometry to identify proteins of *C. trachomatis* and *W. chondrophila* secreted during infection. Among the identified proteins is the Chlamydial Protease-like Activity Factor (CPAF), a well-described secreted protein. Two of the proteins secreted by *C. trachomatis* were ectopically expressed in 293T cells and their subcellular localisation is assessed by immunofluorescence. In addition, two *W. chondrophila* secreted proteins are further characterised with respect to their temporal expression and sub-cellular localization during the course of an infection in Vero cells. 

## 2. Materials and Methods

### 2.1. Cell Culture and Bacterial Strains

Vero, McCoy, HEp-2 and HEK 293T cells (ATCC CCL-81, ECACC 90010305, ATCC CCL-23 and ATCC CRL-3216) were grown at 37 °C, 5% CO_2_ in high glucose Dulbecco’s modified minimal essential medium (DMEM, PAN Biotech, Aidenbach, Germany) supplemented with 10% fetal calf serum (Gibco, Thermo Fisher Scientific, Waltham, MA, USA). *Waddlia chondrophila* strain ATCC VR-1470 was grown at 32 °C within *Acanthamoeba castellanii* strain ATCC 30,010 as described elsewhere [29]. *Chlamydia trachomatis* strain UW-3/Cx Serovar D (ATCC VR-885) was grown in McCoy cells as described in ATCC protocols. 

### 2.2. W. chondrophila Infection Procedure

Vero cells were seeded at 2.5 × 10^5^ cells per well in 24-wells microplates for immunofluorescence or at 4 × 10^6^ cells per 25 cm^2^ flasks for RNA extraction or total protein preparation. Cells were infected the following day with a 1/2000 dilution of *W. chondrophila* recovered from a 4 day-old amoebal co-culture and filtered through a 5 μm filter (Millipore, Carrigtwohill, Ireland) to eliminate amoebal trophozoites and cysts. This bacterial dilution corresponds to an MOI of 2–3, as estimated with a *W. chondrophila* specific real time quantitative PCR [9]. The infection procedure was performed as described in Kebbi-Beghdadi *et al.* [25]. 

### 2.3. C. trachomatis Infection Procedure

For PFO experiments, *C. trachomatis* were grown during 6 days in McCoy cells and recovered by scraping the flask and filtering the lysate through a 5 μm filter. HEp2 cells were infected at an MOI of 10 by centrifuging cells and bacteria during 10 min at 1790 *g* followed by a 15 min incubation at 37 °C. Infected cells were washed 3 times with PBS and further incubated at 37 °C, 5% CO_2_ in DMEM 10% FCS. 

### 2.4. Selective Permeabilisation with Perfringolysin o and Mass Spectrometry Analysis

HEp-2 cells were seeded one day prior to infection in 6-wells microplates at 2 × 10^6^ cells per well and infected with *W. chondrophila* (MOI 10) or *C. trachomatis* (MOI 10) as described above. Twenty-four hours after infection, plates were cooled down on ice during 10 min, washed 3 times with PBS and incubated during 10 min at 4 °C with 100 nM perfringolysin O purified from *E. coli* [30,31] in RPMI medium without phenol red. After three more washes with cold PBS, cells were incubated during 30 min at 37 °C, 5% CO_2_ in 1 mL of RPMI without phenol red medium. Culture supernatant was collected and centrifuged during 3 min at 12,500 *g* before being analysed by shotgun mass spectrometry at the Protein Analysis Facility (PAF) of Lausanne University. Results were analysed by the Facility using the MASCOT software.

### 2.5. Yersinia enterocolitica Type III Secretion Assays

*Y. enterocolitica* HOPEMT (T3SS-proficient) and HOPEMT YscU (T3SS-deficient) strains were used to assess T3SS-dependent secretion, as described in da Cunha et al. [32]. Briefly, full-length genes tagged with a C-terminal V5 epitope were cloned in pLJM3 under control of the *yop* promoter and transformed in the two *Y. enterocolitica* strains. The *E. coli* β lactamase TEM1 gene lacking its predicted N-terminal signal peptide (aa 1–23) was cloned in pLJM3 without a V5 epitope. Bacteria were grown with shaking (200 rpm) in BHI medium supplemented with 20 mM sodium oxalate, 0.4% glucose and 20 mM MgCl_2_ first during 2 h at 27 °C and then at 37 °C in a water bath. This temperature shift activates the *yop* regulon and promotes the secretion of T3SS effectors. After 4 h of growth at 37 °C, absorbance of the culture was measured at OD_600_ and bacteria were pelleted by 1 min centrifugation at 18,000 *g*. Culture supernatants were precipitated over night at 4 °C with trichloroacetic acid. The pelleted proteins from the supernatant were washed with acetone and resuspended in SDS loading buffer. Bacterial pellets were resuspended in SDS loading buffer immediately after centrifugation. Both fractions were normalised according to their OD_600_ values and analysed by SDS-PAGE and immunoblotting

Type III-dependent secretion of Wcw_1706 was also assessed using the *Y. enterocolitica* MRS40 (pNG40031) strain as described in [33]. 

### 2.6. Ectopic Expression in HEK 293T Cells

HEK 293 T cells were seeded on glass coverslips at 1 × 10^5^ cells per well in 24-well microplates. The following day, cells were transfected with pDEST47_CT460/CT432/EfTu or pDEST53 (GFP alone) using Lipofectamine 3000 Reagent (Thermo Fisher Scientific, Waltham, MA, USA) following the manufacturer’s instructions. Twenty-four hours later, cells were fixed with 2% paraformaldehyde during 15 min. Immunofluorescence was performed as described below with a mouse monoclonal anti-GFP antibody (Thermo Fisher Scientific, Waltham, MA, USA) at a 1/500 dilution and DAPI dilactate (4’,6-Diamidino-2-Phenylindole Dihydrochloride, Molecular Probes, Thermo Fisher Scientific, Waltham, MA, USA) at 1/30000 dilution. Cells were observed under a confocal microscope (Zeiss LSM 710 or 780 Quasar Confocal Microscope, Feldbach, Switzerland) at the Cellular Imaging Facility of Lausanne University. 

### 2.7. Purification of Wcw_1706 for Pull Down Experiments and Antibodies Production

The *wcw_1706* full gene was cloned in pCWR547 using NdeI and SacI restriction sites. This plasmid allows addition, at the N-terminal end of the protein of interest, of a 6His tag, followed by a SUMO protease recognition site [34]. Recombinant protein was expressed in BL21 *E. coli* and purified on a Ni-NTA agarose (Quiagen, Hombrechtikon, Switzerland) column under native conditions following the manufacturer’s protocol. After purification, the 6His tag was removed by digestion with SUMO protease (Invitrogen, Thermo Fisher Scientific, Waltham, MA, USA) and the untagged protein was separated from cleaved 6His tag and non-digested 6His_SUMO_Wcw_1706 using Ni-NTA agarose. The non-tagged Wcw_1706 protein was used in pull down experiments and to elicit polyclonal mouse antibodies (Eurogentec, Liège, Belgium).

### 2.8. Pull Down Experiments

*W. chondrophila* T3SS chaperones Wcw_0969, Wcw_0616 and Wcw_0348 were identified by their sequence homology with chlamydial chaperones using ChlamDB [35]. *W. chondrophila* chaperone genes were cloned in pET15b and 6 His-tagged proteins were expressed in *E. coli* BL21 upon addition of 1 mM IPTG to the culture medium. Pull down experiments were performed using the Pierce Pull down PolyHis Protein:Protein Interaction kit (Thermo Fisher Scientific, Waltham, MA, USA) following manufacturer’s instructions. Briefly, His-tagged chaperones recovered from 5 mL *E. coli* cultures were fixed on HisPur Cobalt resin by a 1.5 h incubation at 4 °C in Tris buffer containing 10 mM imidazole. Cultures of non-transformed *E. coli* were used as negative control. After 5 washes in presence of 20 mM imidazole, Cobalt column were incubated during 2 h at 4 °C with purified non-tagged Wcw_1706 protein in phosphate buffer containing 40 mM imidazole and 500 mM NaCl. Columns were washed 5 times with wash buffer containing 20 mM imidazole and protein complexes were eluted 2x from the Cobalt resin using 290 mM imidazole. 

Alternatively, a GST-tagged version of Wcw_1706 was produced in *E. coli* BL21 upon addition of 1 mM IPTG to the culture medium. GST pull down experiments were performed with GST-tagged_Wcw_1706 and His_tagged chaperones Wcw_0969, Wcw_0616 and Wcw_0348 using the Pierce™ GST Protein Interaction Pull-Down Kit (Thermo Fisher Scientific, Waltham, MA, USA), following the manufacturer’s instructions. 

All fractions were analysed by SDS-PAGE and immunoblotting.

### 2.9. Immunoblots

#### 2.9.1. *Y. enterocolitica* Secretion Assays

TCA-precipitated supernatant fractions and pellets were analysed by SDS-PAGE and immunoblotting as described in Kebbi-Beghdadi et al. [36]. Proteins of interest were detected using a mouse monoclonal anti-V5 epitope antibody (Invitrogen, Thermo Fisher Scientific, Waltham, MA, USA) (1/5000 dilution) or anti β lactamase antibody (VWR International GmbH, Dietikon, Switzerland) (1/1000 dilution) and MreB was detected using a home-made rabbit polyclonal antibody (1/5000 dilution). First antibodies were incubated overnight at 4 °C. Secondary antibodies, goat anti-mouse IgG-HRP (BioRad, Cressier, Switzerland) and donkey anti-rabbit IgG-HRP (Promega, Dübendorf, Switzerland), both diluted 1/3000 were applied during 1 h at room temperature. Immunoblots were revealed with ECL^TM^ Prime Western Blotting Detection Reagent (Amersham, GE Healthcare, Glattbrugg, Switzerland) and analysed on ImageQuant LAS4000 mini (Amersham, GE Healthcare, Glattbrugg, Switzerland). 

#### 2.9.2. *W. chondrophila* Infected Cells

*W. chondrophila-*infected Vero cells and culture supernatants were harvested at different time points after infection and DNA was extracted from a 50 μL aliquot using Wizard SV genomic DNA extraction kit (Promega, Dübendorf, Switzerland). Bacteria were quantified using a *W. chondrophila* specific qPCR [9] as described in Kebbi-Beghdadi et al. [25]. Cells and bacteria from one T25 flask were pelleted by 5 min centrifugation at 12,000*g*. Pellets were washed with PBS and resuspended in 500 μL of SDS-PAGE loading buffer. Proteins were analysed by SDS-PAGE and immunoblotting as described in Kebbi-Beghdadi et al. [36]. Wcw_1706 was detected with a polyclonal mouse anti-Wcw_1706 antibody diluted 1/1000 in TBS with 0.05% Tween 20 and 5% non-fat dry milk and incubated overnight at 4 °C. Goat anti-mouse IgG-HRP conjugated antibodies (BioRad, Cressier, Switzerland (1/3000 dilution) were applied during 1 h at room temperature. Immunoblots were revealed with ECL^TM^ Prime Western Blotting Detection Reagent (Amersham, GE Healthcare, Glattbrugg, Switzerland) and images acquired on ImageQuant LAS4000 mini (Amersham, GE Healthcare, Glattbrugg, Switzerland). Signal intensities were measured with ImageJ, normalised according to the number of bacteria determined by quantitative PCR and expressed as a percentage of the maximum value.

#### 2.9.3. Pull Down Experiments

All fractions were analysed by SDS-PAGE and immunoblotting as described in Kebbi-Beghdadi et al. [36]. The mouse polyclonal anti_Wcw_1706 antibody diluted 1/1000 was incubated overnight at 4 °C in TBS with 0.05% Tween 20 and 5% non-fat dry milk. Mouse monoclonal anti-GST (Sigma-Aldrich, Buchs, Switzerland) and anti-His (Sigma-Aldrich, Buchs, Switzerland) antibodies were diluted respectively 1/1000 or 1/3000 and incubated during 2 h at room temperature in TBS with 0.05% Tween 20 and 0.5% non-fat dry milk. Secondary goat anti-mouse IgG-HRP (BioRad, Cressier, Switzerland) antibody (1/3000 dilution) was applied during 1 h at room temperature. Immunoblots were revealed with ECL^TM^ Western Blotting Detection Reagent (Amersham, GE Healthcare, Glattbrugg, Switzerland) and analysed on ImageQuant LAS4000 mini (Amersham, GE Healthcare, Glattbrugg, Switzerland). 

### 2.10. Gene Expression

*W. chondrophila-*infected Vero cells and culture supernatants were collected in TRIzol (AmbionR, Life Technologies, Thermo Fisher Scientific, Waltham, MA, USA) at different time points after infection. RNA was extracted as described in Chomczynski and Mackey [37]. Random primers and the GoScript Reverse Transcription kit (Promega, Dübendorf, Switzerland) were used to produce cDNA that was analysed by quantitative PCR using I Taq SYBRGreen technology (BioRad, Cressier, Switzerland). qPCR were performed with 4 μL of cDNA diluted 1/5 and 300 nM (16S and wcw_0499) or 200 nM (wcw_1706) of the following primers:

16S for: 5′ GGCCCTTGGGTCGTAAAGTTCT 3′
16S rev: 5′ CGGAGTTAGCCGGTGCTTCT 3′
wcw_1706 for: 5’ TTGACGCTTGTCGAGGTTCA 3’
wcw_1706 rev: 5’ GCAAAAACTCCGGCACTTCC 3’
wcw_0499 for: 5’ TGTGCGTGAGTTTTCAGAGGA 3’
wcw_0499 rev: 5’ TTTATTGGTTTGCAGGGCGC 3’

Cycling conditions were 10 min at 95 °C, followed by 40 cycles of 15 s at 95 °C and 1 min at 60 °C. PCR products were amplified and detected with the StepOne Real-Time PCR System (Applied Biosystems, Zug, Switzerland). qRT-PCR results were analysed using 16S rRNA gene as an endogenous control and 48 h pi as the reference time point. 

### 2.11. Immunofluorescence and Confocal Microscopy

Vero cells were grown during 16 h on glass coverslips and infected with *W. chondrophila* as described above. At 8, 16, 24 and 32 h post infection, cells were fixed with ice-cold methanol during 5 min at –20 °C, washed three times with PBS and incubated at least 2 h in blocking solution (PBS, 0.01% NaN_3_, 1% BSA) at 4 °C. Coverslips were incubated with a mouse anti-Wcw_1706 antibody diluted 1/1000 in PBS, 0.1% saponin, 1% BSA or with a mouse antibody elicited against a Wcw_0499 peptide (aa 128 to 141) diluted 1/500 in the same solution. First antibodies were applied during 2 h at room temperature. After three washing steps in PBS, 0.1% saponin, coverslips were incubated with a 1/500 dilution of AlexaFluor 488-conjugated goat anti-mouse antibody (Life Technologies, Thermo Fisher Scientific, Waltham, MA, USA) and a 1/30000 dilution of DAPI dilactate (4’,6-Diamidino-2-Phenylindole Dihydrochloride, Molecular Probes, Thermo Fisher Scientific, Waltham, MA, USA). Secondary antibody was applied during 1 h at room temperature. After 2 washes with PBS 0.1% saponin, 1 with PBS and 1 with deionised water, the coverslips were mounted onto glass slides using Mowiol (Sigma-Aldrich, Buchs, Switzerland). Cells were observed under a confocal microscope (Zeiss LSM 710 or 780 Quasar Confocal Microscope, Feldbach, Switzerland) at the Cellular Imaging Facility of Lausanne University.

## 3. Results

### 3.1. Selective Eukaryotic Membrane Permeabilisation Identifies Secreted Bacterial Proteins

Intracellular bacteria secrete effectors in the host cell cytosol to modulate their environment and create favourable conditions to complete their life cycle. To identify bacterial proteins present in host cell cytosol, we used perfringolysin O (PFO), an exotoxin produced by *Clostridium perfringens* that forms pores in cholesterol-containing membranes. 

Perfringolysin O was previously demonstrated to allow transient permeabilisation of the eukaryotic plasma membrane without disruption of the inclusion membrane integrity [31]. We analysed by mass spectrometry the culture medium of perfringolysin O-treated HEp2 cells infected with *C. trachomatis* or *W. chondrophila.* This fraction contains the cytosolic proteins, including host cell proteins, as well as proteins secreted by bacteria. Infected but non-permeabilised cells were used as control to take account of bacteria still present in the culture medium after the infection procedure and of putative host cell lysis. All bacterial proteins identified in the PFO-treated sample and absent in mock-treated control as well as those enriched more than 1.5 times in the PFO-treated sample versus the mock control are presented in Supplementary Table S1. This list includes several proteins, such as chaperones (DnaK, GroL, GroS), ribosomal proteins (50S ribosomal proteins L7/L12, L9 and L10, 30S ribosomal protein S6) or elongation and initiation factors (Elongation factors Tu, Ts and G, Translation initiation factor IF-1) that are repeatedly retrieved in mass spectrometry analyses performed on different fractions containing *W. chondrophila* proteins [36,38,39]. They are probably very abundant proteins and likely represent technical contaminants. For the sake of simplification, they were discarded from the results of Table 1 that presents proteins presumably secreted by *C. trachomatis* or by *W. chondrophila* in the cytosol of their host cell. 

Interestingly, the Chlamydial Protease-like Activity Factor (CPAF) was retrieved in the cytosol of cells infected with *C. trachomatis* (CT858) or with *W. chondrophila* (Wcw_0991). This protein is an important virulence factor detected in the host cell cytoplasm in several studies [40,41,42,43]. Furthermore, in the analyses of cells infected with *C. trachomatis,* we retrieved with high confidence the Chlamydia protein Associating with Death Domains (CADD) (CT610), a protein secreted by the Type III Secretion System (T3SS) and capable of interacting with mammalian death receptors [44,45]. The recovery of these known secreted proteins in our analyses validates the use of the PFO permeabilisation method to identify new bacterial effector proteins secreted in host cell cytosol. Although in the case of intracellular bacteria Sec-dependent secretion systems usually deliver proteins in the lumen of the bacteria-containing vacuole (i.e., in the lumen of the inclusion for *Chlamydiales* bacteria), effectors can also travel from the inclusion lumen to the host cell cytosol through outer membrane vesicles [19,46]. Interestingly 3 out of 13 (23%) proteins identified in the cytosol of cells infected with *C. trachomatis* display a predicted signal peptide, while it is the case for 13 out of 19 (68%) in *W. chondrophila*-infected cells. 

*Chlamydiales* bacteria also possess a T3SS able to secrete virulence factors directly into the host cell cytosol. Seven out of ten proteins without a predicted N-terminal signal peptide identified in the experiment with *C. trachomatis* and 3 out of 6 identified in the experiment with *W. chondrophila* are predicted to be T3SS effectors by at least one algorithm trained in silico to recognise such secreted proteins [35]. 

Altogether, a large majority of the proteins identified with the PFO permeabilisation protocol are predicted to be secreted proteins, which confirms the relevance of this technique for the identification of new bacterial effector proteins. 

### 3.2. Four Newly Identified Putative Chlamydial Effector Proteins Are Secreted by Yersinia enterocolitica T3SS

**Figure 1:**
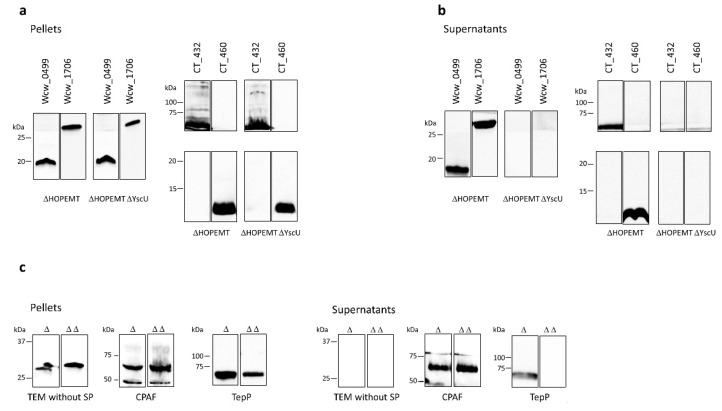
T3SS-dependent secretion in *Y. enterocolitica.* The putative secreted proteins Wcw_0499, Wcw_1706, CT432 and CT460 tagged with a V5 epitope were detected by immunoblot in the bacterial pellet (**a**) or in the culture supernatant (**b**) of *Y. enterocolitica* T3SS-proficient (ΔHOPEMT) and T3SS-deficient (ΔHOPEMT ΔYscU) strains. (**c**) Control experiments were performed with TEM without signal peptide (no secretion), CPAF (secreted by T2SS [43]) and TepP (secreted by T3SS [50,51]). For each protein, result of one representative experiment is presented in the figure but all experiments were repeated 3 times independently with congruent results. Blots were cropped and grouped, see Supplementary Figures S1–S3 for complete blot pictures.

Members of the *Chlamydiales* order have been historically recalcitrant to genetic modifications. During the last decade, a few systems were developed to manipulate genetically *C. trachomatis* but *Chlamydia*-related bacteria remain intractable for now [49]. Heterologous systems are thus mandatory to study chlamydial genes and pathways and in particular the T3SS. In this study, we used *Yersinia enterocolitica* to assess T3SS-dependent secretion of four putative secreted proteins identified by PFO permeabilisation: CT460, CT432, Wcw_0499 and Wcw_1706. These proteins are predicted in silico to be T3SS effectors by one algorithm trained to identify such secreted proteins (T3_MM model [50] for CT432, CT460, Wcw_1706 and effective T3 [51] for Wcw_0499) and they do not possess a predicted N-terminal signal peptide [35]. The four proteins tagged with a V5 epitope were expressed in *Y. enterocolitica* T3SS-proficient (HOPEMT) and T3SS-deficient (HOPEMT YscU) strains under the control of the *Yersinia* y*opE* gene promoter [32]. We analysed by immunoblot the presence of the tagged proteins in the bacterial pellets and in the culture supernatants from both strains. As depicted on Figure 1a, efficient expression of the four proteins in both T3SS-proficient and T3SS-deficient strains was validated by their detection in the bacterial pellets. In addition, all four proteins were present in the culture medium of the T3SS-proficient strain and absent in the supernatant of the T3SS-deficient one (Figure 1b). Interestingly, the secreted form of Wcw_0499 was slightly smaller than the form detected in the pellet fraction. This size shift was observed consistently in every experiment performed with this protein and probably indicates a proteolytic cleavage that could be associated with an activation of the secreted form. 

Three proteins were used as controls in this assay (Figure 1c): (i) a truncated form of the *E. coli* β lactamase lacking its signal peptide (TEM without SP) that should not be secreted, (ii) CPAF, a protein secreted by a Sec-dependent pathway [46] and (iii) the *C. trachomatis* translocated early phosphoprotein, TepP (CT875), a known T3SS effector [52,53]. These three control proteins were detected in the bacterial pellets of both *Y. enterocolitica* strains, demonstrating efficient expression. TEM without SP was not present in the culture supernatant of either strain, whereas CPAF was detected in both HOPEMT and HOPEMT YscU supernatants. TepP was detected only in the case of a functional T3SS. To exclude bacterial lysis or fraction contamination, the supernatant fractions were probed against the strictly cytosolic *Y. enterocolitica* MreB protein (Supplementary Figures S1–S3) [33]. 

In addition, T3SS-dependent secretion of Wcw_1706 was also convincingly demonstrated using a third *Y. enterocolitica* strain in which secretion depends on the presence of calcium in the culture medium (Supplementary Figure S4 [33,54]). 

Altogether, our results demonstrated that the four putative secreted effector proteins that we selected for analysis in the *Y. enterocolitica* system are indeed secreted by a heterologous T3SS, thus validating at once the relevance of the PFO permeabilisation technique to uncover new T3SS effectors and the significance of algorithms to predict such effectors. 

### 3.3. CT460 Localises in Host Cell Nucleus upon Transfection

**Figure 2:**
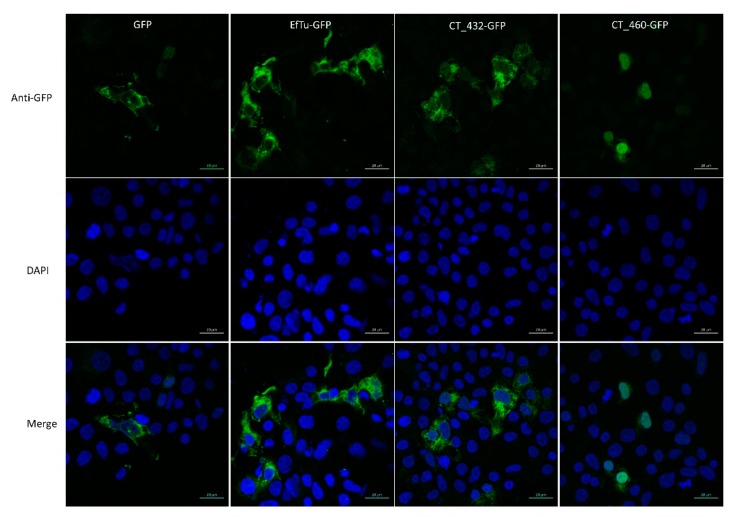
CT460 localises in host cell nucleus upon transfection. 293T cells transfected with GFP-tagged CT432 or CT460 were stained with anti-GFP antibody (green) and DAPI (blue). GFP-tagged EfTu and GFP alone were used as negative controls. Scale bar: 20 μm.

The putative T3SS effector CT460 contains a SWIB/MDM2 domain potentially involved in chromatin remodelling. Furthermore, the *W. chondrophila* homolog of this protein, Wcw_0377, was recently demonstrated to be associated with bacterial DNA in infected Vero cells and to localise in the host nucleus in transfected 293T cells [55]. We wondered if a similar nuclear localisation could be observed with CT460. To answer this question, we transfected 293T cells with a GFP-tagged version of CT460 and CT432, a serine hydroxymethyltransferase that is presumably not associated with nuclei, and we analysed their localisation by immunofluorescence. A *W. chondrophila* cytosolic protein, Elongation factor Tu (EfTu) fused to GFP and GFP alone were used as negative controls in these experiments. As shown in Figure 2, the green signal corresponding to CT460-GFP clearly co-localised with the DAPI staining of nucleus in transfected cells, while CT432-GFP and the two negative control proteins were mainly localised in 293T cell cytoplasm. Similar results were obtained with the GFP tag located at the C-terminus or at the N-terminus of CT460. These results suggest that, similarly to the T3SS effector NUE [56], CT460 could localise in the nucleus of infected cells and play a role in chromatin remodelling. Interestingly, we could also demonstrate that the *W. chondrophila* homolog of CT460, Wcw_0377, is secreted by the *Y. enterocolitica* T3SS (data not shown), which further supports the putative secretion of these two proteins through chlamydial T3SS during infection and emphasises the efficiency of the PFO method to discover new potential effectors. 

### 3.4. Wcw_0499 Is an Early to Mid-Cycle Effector Protein 

*W. chondrophila* has a much broader host range than *C. trachomatis* and probably encodes a larger number of effectors specifically associated with this distinctive characteristic. Thus, this *Chlamydia*-related bacterium represents an interesting model to study how *Chlamydiales* bacteria manipulate their host cell and which strategies they have evolved to survive in diverse environments. In this respect, we further characterised two *W. chondrophila* proteins of unknown function, Wcw_0499, that is strictly specific to *W. chondrophila*, and Wcw_1706, a protein with homologs in all other species of the *Chlamydiales* order [35]. 

Previously published studies by our group showed that in Vero cells EBs differentiate into RBs as early as 3 h post infection (pi). Exponential multiplication takes place between 8 and 32 h, then RBs asynchronously re-differentiate into EBs that are released by host cell lysis. In this cell line, *W. chondrophila* completes its replication cycle in about 48 h [25,36]. 

**Figure 3:**
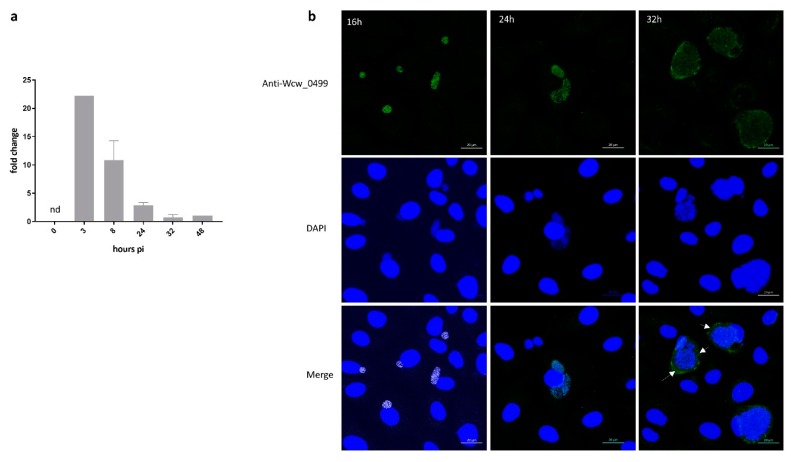
Wcw_0499 is an early to mid-cycle effector detected in the host cell cytosol at late stages of infection (**a**) *Wcw_0499* transcriptional expression was analysed by RT-qPCR during the course of an infection in Vero cells and normalised at 48h pi according to 16SrRNA gene expression. Results are the mean and SD of three independent experiments. At 3 h post infection, expression could only be detected in one experiment. nd: not detected. (**b**) Vero cells infected with *W. chondrophila* were stained with a polyclonal mouse antibody against Wcw_0499 (green) and DAPI (blue) at 16, 24 and 32 h post infection. White arrows point at area where secretion can be observed. Scale bar: 20μm.

To assess the transcriptional profile of *wcw_0499* gene during a complete *W. chondrophila* replication cycle, we extracted RNA from *W. chondrophila*-infected Vero cells at different time points during the course of infection. We performed RT-qPCR using the 16SrRNA gene as an internal reference and normalised the results to *wcw_0499* gene expression at 48 h pi. Data presented in Figure 3a indicate that the *wcw_0499* gene is transcribed very early during the developmental cycle of the bacteria, with a peak of transcription detected at 3 h pi. 

We then analysed by immunofluorescence the expression and localisation of Wcw_0499 during the course of a *W. chondrophila* infection in Vero cells. The signal corresponding to Wcw_0499 co-localised with the DAPI staining of growing bacteria (RBs) but could be detected only from 16 h pi onwards (Figure 3b), which suggests that Wcw_0499 is weakly expressed. As can be seen in Figure 3b, the signal corresponding to Wcw_0499 precisely co-localised with DAPI staining of bacterial DNA at 16 and 24 h pi. At 32 h pi, the Wcw_0499 specific signal was less strongly staining bacteria and could also be detected outside the inclusion, in host cell cytoplasm, suggesting a possible secretion (Figure 3b, white arrows). This observation is consistent with previous results (Table 1), showing that Wcw_0499 could be retrieved in the host cell cytoplasm but should be taken cautiously since secretion is difficult to observe at such late time points when large inclusions occupy most of the eukaryotic cell cytoplasm. Nevertheless, our results indicate that Wcw_0499 is an early to mid-cycle effector protein, detected in the host cell cytosol at late stages of infection.

### 3.5. Wcw_1706 Is a Mid-Cycle Effector Protein Interacting with a T3SS Class II Chaperone

**Figure 4:**
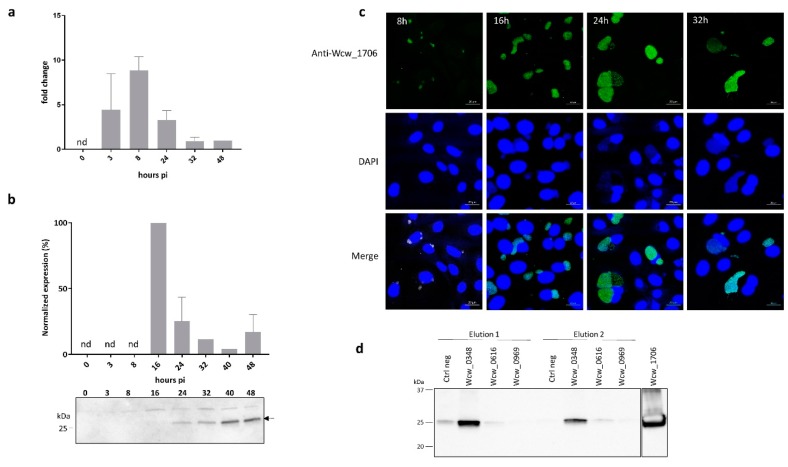
Wcw_1706 is a mid-cycle effector protein interacting with a T3SS class II chaperone (**a**) *wcw_1706* transcriptional profile was analysed by RT-qPCR during the course of an infection in Vero cells and normalised at 48h pi according to 16SrRNA gene expression. Results are the mean and SD of three independent experiments. nd: not detected. (**b**) Wcw_1706 expression was monitored by immunoblot during the course of an infection in Vero cells. Arrow indicates the band corresponding to Wcw_1706. Full-length immunoblot is presented in Supplementary Figure S5. The signal intensity measured with ImageJ was normalised according to the number of bacteria in the sample and expressed as percentage of the maximum value. Results are the mean and SD of three independent experiments. nd: not detected. (**c**) Vero cells infected with *W. chondrophila* were stained with a polyclonal antibody against Wcw_1706 (green) at 8, 16, 24 and 32 h post infection. DNA is stained with DAPI (blue). Scale bar: 20 μm. (**d**) Immunoblot analysis of elution fractions 1 and 2 from a pull down experiment with Wcw_1706 and the three T3SS chaperones Wcw_0969, Wcw_0616 and Wcw_0348. Negative control = no protein. A mouse polyclonal anti-Wcw_1706 antibody was used to probe the blot. Full-length immunoblot is presented in Supplementary Figure S6. Experiments were repeated three times independently with congruent results.

We then focused our interest on Wcw_1706, a protein of unknown function encoded in the genome of all bacteria of the *Chlamydiales* order. The transcriptional pattern of *wcw_1706* gene was determined by RT-qPCR at different time points following infection of Vero cells with *W. chondrophila* and the profile presented in Figure 4a indicates that *wcw_1706* is transcribed early during the developmental cycle, with a peak of transcription detected at 8 h pi. 

Protein expression was analysed in infected cells by immunoblot using a specific anti-Wcw_1706 antibody. Relative abundance of the protein was quantified by normalising the signal measured on the blot according to the number of bacteria loaded in each lane as assessed by quantitative PCR [9]. The highest protein expression (defined as 100%) was observed at 16 h pi but the low sensitivity of this technique did not allow detection before this time point (Figure 4b). Protein expression could also be visualised by immunofluorescence staining of infected Vero cells. This technique allowed detection of a signal corresponding to Wcw_1706 already at 8 h pi (Figure 4c). At this and later time points, the specific signal corresponding to Wcw_1706 nicely co-localised with the DAPI staining of bacterial DNA in inclusions. 

Most T3SS effectors require specific bacterial chaperones for proper folding and delivery into host cell cytosol. We assessed by pull down experiments the binding of Wcw_1706 to three *W. chondrophila* T3SS chaperones that were identified by sequence homology to known chlamydial chaperones. Wcw_0969 is homologous to SlcI (61% sequence identity), Wcw_0616 is the *W. chondrophila* counterpart of Multiple Cargo Secretion Chaperone (Mcsc) (29% sequence identity) and Wcw_0348, a protein with a tetratricopeptide repeat-containing domain, is homologous to class II chaperone CT274 (51% sequence identity) [21,57,58]. Elution fractions of a pull down experiment with Wcw_1706 and the three above-mentioned *W. chondrophila* chaperones were analysed by immunoblot using anti-Wcw_1706 antibody. As shown in Figure 4d, no specific interaction could be detected between Wcw_1706 and chaperones Wcw_0969 and Wcw_0616. Wcw_1706 was recovered only in pull down experiments with chaperone Wcw_0348 indicating a clear interaction between this T3SS effector and the class II chaperone. This result was further confirmed using a GST-tagged version of 1706 (Supplementary Figure S7). The interaction of Wcw_1706 with a T3SS chaperone further supports the results obtained in the *Y. enterocolitica* secretion assay, suggesting that this protein is a T3SS effector.

## 4. Discussion

In this study, we used the pore-forming toxin perfringolysin O to selectively permeabilise the plasma membrane of eukaryotic infected cells without disrupting the inclusion membrane. This protocol, described by Kleba and Stephens in 2008, allowed a mass spectrometry analysis of the culture medium containing all cytosolic proteins, including secreted bacterial effectors, but with a limitation to soluble and relatively small proteins. Effectors that would bind to membrane proteins or interact in complexes with host cell components for example could remain trapped within the cell during PFO treatment. Twenty-one *C. trachomatis* and 28 *W. chondrophila* proteins were identified in this analysis. Among them are abundant proteins such as chaperones, ribosomal proteins or elongation and initiation factors that are repeatedly retrieved in *W. chondrophila* mass spectrometry analyses [36,38,39] and thus likely represent contaminant abundant proteins. The remaining 13 *C. trachomatis* and 19 *W. chondrophila* putative secreted proteins include the Chlamydial Protease-like Activity Factor (CPAF), that was retrieved in screenings with both bacteria, and the *Chlamydiaceae* specific CADD (Chlamydia protein Associating with Death Domains). Secretion was previously demonstrated for these two proteins [42,45]. CPAF is a protease exhibiting a predicted N-terminal signal peptide. It is first secreted in the inclusion lumen by the T2SS, and later reaches the host cell cytosol by a yet unclear mechanism. It modulates several host cell pathways to evade the innate immune response and promote bacterial survival (reviewed in [20]). CADD is secreted by the T3SS into the host cytoplasm, where it co-localises with tumour necrosis factor receptors and interacts with their death domains to modulate apoptosis pathways [45]. On the opposite, CT539, a thioredoxin involved in the reduction of oxidized cysteine residues and the cleavage of disulphide bonds, was reported by Wu et al. not to be secreted in the host cell cytoplasm using conventional fluorescence microscopy [45]. The discrepancy between these results and our data could be due to different sensitivities of the technique used, MS analyses being far more sensitive than immunofluorescence. On this point, it is worth noting that in a list of 30 effector proteins of *C. trachomatis* published by Bugalhao and Mota [20], only one third were observed in the host cell cytoplasm using immunofluorescence [46,48,59,60,61,62,63,64,65]. Alternatively, CT539 could also be a very abundant but not secreted protein contaminating our results as described above for ribosomal proteins. 

Remarkably, 8 out of 13 *C. trachomatis* putative effectors identified with the PFO permeabilisation protocol and 16 out of 19 *W. chondrophila* putative effectors are predicted in silico to be T3SS effectors or contain a N-terminal signal peptide for the general secretory pathway, or both. Our experiments using the heterologous system *Y. enterocolitica* to assess T3SS-dependent secretion of four proteins predicted in silico to be T3SS substrates demonstrated that algorithms are indeed able to predict secretion by the T3SS. Thus, it is reasonable to hypothesise that proteins in Table 1 predicted to be T3SS effectors based on computer analyses are likely to be secreted in the host cell cytosol. On the other hand, the Sec system translocates into the periplasmic space proteins that can further be exported in the inclusion lumen via the T2SS or outer membrane vesicles (OMVs). These vesicles will ultimately fuse with or pass through the inclusion membrane liberating the proteins in the host cell cytosol [20,46]. Indeed, CPAF (that possesses a signal peptide) was detected experimentally in host cytosol in several independent studies [40,41,42,43]. We can reasonably assume that other proteins exhibiting a predicted signal peptide might also be secreted within the host cell cytosol. OMVs consist of periplasmic content, membrane-bound proteins, lipopolysaccharides and phospholipids [66]. The identification in the cytosol of cells infected with *C. trachomatis* or with *W. chondrophila* of a Skp-like protein that can interact with membrane lipids and lipopolysaccharides is in line with this explanation [67]. 

The eight proteins found secreted in the present study, which do not harbor a N-terminal signal peptide and are not predicted in silico to be T3SS effectors could have reached the host cytoplasm in association with other proteins or by yet unknown mechanisms. 

In agreement with their detection in the host cell cytoplasm, 5 out of 13 *C. trachomatis* putative secreted proteins (CT610, CT112, CT242, CT603 and CT507) were previously demonstrated to be immunogenic proteins [47,68,69,70]. After host cell lysis, these proteins are probably released in the extracellular space where they can be recognized by the immune system. 

As inferred from their putative function, several of the newly identified putative secreted effectors could interfere with cellular metabolic pathways and host defence mechanisms to bacterial benefit. CT460 is of particular interest given its putative chromatin remodelling SWIB/MDM2 domain. Wcw_0377, the *W. chondrophila* homolog of CT460 possesses the same SWIB/MDM2 domain and was recently demonstrated to reach the host cell nucleus where it could interact with eukaryotic histones [53]. The SWIB/MDM2 domain is mainly present in eukaryotic proteins and rarely found in bacterial proteins [35], suggesting that proteins containing such domains are likely to be implicated in bacteria-host interactions. Our results using the *Y. enterocolitica* secretion assay demonstrated that CT460 as well as Wcw_0377 are T3SS substrates. In addition, ectopic transfection in 293T cells indicated a nuclear localisation of these two proteins (this study and [53]). These findings suggest that CT460/Wcw_0377 could be nuclear effectors possibly exhibiting a methyltransferase activity towards eukaryotic histones similarly to NUE, a T3SS effector that localises in the nucleus of infected cells and is thought to methylate histones and remodel chromatin [54]. 

*C. trachomatis* and *W. chondrophila* belong to the same phylogenetic order and share several important features of strict intracellular bacteria. In this respect, it is worth noting that two proteins, CPAF (CT858 and Wcw_0991) and the Skp-like protein OmpH (CT242 and Wcw_1192), were identified both in the experiments with *C. trachomatis* and with *W. chondrophila*. This interesting result suggests that some eukaryotic pathways important for bacterial survival and replication are targeted similarly by all member of the *Chlamydiales* order. On another hand, *C. trachomatis* and *W. chondrophila* display large differences in their host range and intracellular trafficking that are very likely partly due to a distinct arsenal of T3SS effectors. *W. chondrophila* also possesses a much larger genome than *C. trachomatis* and consequently encodes potentially many more effectors [71]. Thus, studying *W. chondrophila* specific effectors could provide great opportunities to understand the strategies evolved by the different members of the *Chlamydiales* order to survive in their diverse host environments. Indeed, among the bacterial proteins identified in the cytosol of *W. chondrophila*-infected Vero cells, three hypothetical proteins Wcw_0499, Wcw_0680 and Wcw_0704 are only present in *W. chondrophila* and are thus of particular interest. 

The transcription profile of *wcw_0499* revealed that this gene is transcribed at the very beginning of the developmental cycle suggesting that the corresponding protein could interfere with early mechanisms of host cell response to infection. This protein could be linked to the ability of *W. chondrophila* to survive in different cells including amoebae and macrophages, an ability probably related to the rapid escape of the bacterium from the endocytic pathway [16]. Alternatively, Wcw_0499 could be also implicated in the recruitment of mitochondria around the *W. chondrophila* inclusion, an event that occurs very rapidly after bacterial entry in the host [16]. Determining the host cell component(s) interacting with Wcw_0499 will help resolving this question und unravel part of the chlamydial evolution and virulence. 

Our immunofluorescence experiments suggested secretion of Wcw_0499 in Vero cell cytosol at 32 h pi. No secretion could be observed at earlier time points even though this protein was identified in the cytosol of infected HEp2 cells 24 h pi. This difference could be due to a different timing of *W. chondrophila* replication cycle in these two cell lines. It could also reflect a technical limitation in the detection by immunofluorescence of small amount of protein secreted in the host cell cytosol. Indeed, as mentioned above, chlamydial secreted effector proteins are notably difficult to observe by confocal microscopy in the cytosol of infected cells, certainly due to their low amount and to their transient time of secretion. Wcw_1706 is a hypothetical protein of unknown function identified as immunogenic in a previous work by Lienard et al. [39]. It is a *Chlamydiales*-specific protein, conserved throughout the phylum and presenting about 50% sequence homology with its chlamydial homologs such as CT538 and Cpn_0658 [72]. We demonstrated in the present study that a full-length version of Wcw_1706 is secreted by the T3SS of *Y. enterocolitica*. However, two previously published studies indicated that CT538 and Cpn_0658 are not T3SS substrates [32,73]. These results were obtained in secretion assays performed with the N-terminal part of the protein (about 40 aa) fused to a reporter gene in *Yersinia* or *Shigella* heterologous systems. Since CT538 and Cpn_0658, that have 77% amino acids identity in their N-terminal region, share only 40% identity with Wcw_1706 in this same region, it is not surprising that Wcw_1706 behaves differently in the *Yersinia* secretion assay. 

Nothing is known about the function of Wcw_1706 or its chlamydial homologs and no clue can be inferred from their amino acid sequences. However, our results demonstrated that *wcw_1706* transcription is maximal at 8 h pi, when exponential bacterial multiplication starts within an inclusion that is already tightly associated with mitochondria and localised in the endoplasmic reticulum [16]. This expression profile suggests a role for Wcw_1706 in nutrient acquisition or disruption of cellular pathways. 

Finally, CT538, the *C. trachomatis* homolog of Wcw_1706, is one of the 10 iron-responsive proteins identified by Dill et al. [74]. Surprisingly, four other proteins, whose expression was increased when bacteria were grown in iron-restricted versus iron-sufficient conditions (CT507, CT603, CT610 and CT707), were identified in the host cell cytosol in our PFO assay. In addition, transcriptional studies revealed an upregulation of CT112, CT432 and CT505 when infected cells were grown respectively in presence of an iron chelator or of IFN gamma [75,76]. Altogether 7 out of 13 *C. trachomatis* proteins identified in our study seem to be implicated in stress response. In this respect, it would be interesting to explore the potential correlation between the expression of T3SS effector proteins and response to stress conditions. These secreted proteins being very important in the molecular interplay between bacteria and host cells, they deserve a particular interest in the context of nutrient acquisition as well as persistence of *Chlamydiales* bacteria.

To conclude, this work reports a screening for secreted proteins of *C. trachomatis* and *W. chondrophila* using PFO to gain access to the protein content of infected cells cytosol. This screening enabled the identification of several interesting *C. trachomatis* putative effectors potentially involved in chromatin remodelling or in responses to stressful conditions. In addition, we also shed light on two *W. chondrophila* T3SS effectors, Wcw_0499 and Wcw_1706 that are implicated in the early to mid-stages of infection. Determining the cellular pathways targeted by these proteins will certainly help understanding the different strategies evolved by *Chlamydiales* bacteria to survive in their diverse environments. 

## Figures and Tables

**Table 1:** Proteins presumably secreted by *C. trachomatis* or by *W. chondrophila* in their host cell cytosol.

Gene Number	Identified Proteins	Gene	Molecular	Signal	T3SS Effectors	Previously Described
		Name	Weight	Peptide	Prediction ^1^	as
***C. trachomatis***						
CT_610	PqqC-like protein (CADD)		27 kDa	NO	0	secreted [45]
CT_460	SWIB (YM74) complex protein		10 kDa	NO	1	nd
CT_112	Oligoendopeptidase	*pepF*	69 kDa	NO	1	nd
CT_539	Thioredoxin	*trxA*	11 kDa	NO	1	not secreted [47]
CT_771	Hydrolase/phosphatase homolog		17 kDa	NO	0	nd
CT_242	Skp-like protein		19 kDa	YES	1	nd
CT_707	Trigger factor	*tig*	50 kDa	NO	2	nd
CT_432	Serine hydroxymethyltransferase	*glyA*	54 kDa	NO	1	nd
CT_691	Hypothetical protein		25 kDa	YES	1	nd
CT_505	Glyceraldehyde-3-phosphate dehydrogenase	*gapA*	36 kDa	NO	0	nd
CT_603	Thio-specific Antioxidant (TSA) Peroxidase	*ahpC*	22 kDa	NO	0	nd
CT_858	Chlamydial protease-like activity factor	*cpaf*	67 kDa	YES	0	secreted [42,48]
CT_507	DNA-directed RNA polymerase subunit alpha	*rpoA*	42 kDa	NO	0	nd
***W. chondrophila***						
wcw_0501	Hypothetical protein		47 kDa	YES	3	nd
wcw_0432	DO serine protease	*htrA3*	52 kDa	YES	0	nd
wcw_0499	Hypothetical protein		18 kDa	NO	1	secreted (this study)
wcw_1545	Nucleoside diphosphate kinase	*ndk2*	18 kDa	YES	0	nd
wcw_0991	Putative chlamydial protease-like activity factor	*cpaf*	67 kDa	YES	0	nd
wcw_1192	Putative Skp-like protein	*ompH*	21 kDa	YES	1	nd
wcw_1543	Nucleoside diphosphate kinase	*ndk1*	16 kDa	NO	0	nd
wcw_0657	Hypothetical protein		26 kDa	YES	0	nd
wcw_0453	Putative rhs family protein	*rhs11*	200 kDa	YES	0	nd
wcw_0967	Hypothetical protein		31 kDa	YES	0	nd
wcw_0878	NADPH-dependent FMN reductase		23 kDa	YES	0	nd
wcw_1529	Peptidyl-prolyl cis-trans isomerase	*mip3*	28 kDa	YES	0	nd
wcw_0704	Hypotheticalprotein		526 kDa	NO	1	nd
wcw_1706	Hypothetical protein		28 kDa	NO	1	secreted (this study)
wcw_0680	Hypothetical protein		40 kDa	YES	0	nd
wcw_0715	RNA-binding protein	*rbp*	10 kDa	NO	0	nd
wcw_0969	Hypothetical protein		18 kDa	NO	0	nd
wcw_1068	Peptidyl-prolyl cis-trans isomerase	*ppiB*	22 kDa	YES	0	nd
wcw_1301	Hypothetical protein		17 kDa	YES	1	nd

Bacterial proteins identified by mass spectrometry analysis in the culture medium of perfringolysin O-treated HEp2 cells infected with *C. trachomatis* or *W. chondrophila.* nd: not determined ^1^ Number of algorithms predicting secretion by T3SS (out of 4) [35].

## Data Availability

The datasets generated and/or analysed during the current study are available from the corresponding author on reasonable request.

## Supplementary Materials

The following are available online at https://www.mdpi.com/2076-2607/8/3/361/s1.
