# Supplementary material for: Eukaryotic Cell Permeabilisation to Identify New Putative Chlamydial Type III Secretion System Effectors Secreted within Host Cell Cytoplasm
Source: Microorganisms. 2020 Mar 3;8(3):361. doi: 10.3390/microorganisms8030361 (PMC7143554; doi:10.3390/microorganisms8030361)
Supplement: Supplementary file 1 [file microorganisms-08-00361-s001.pdf]

Eukaryotic cell permabilisation to identify new putative chlamydial Type III Secretion System effectors secreted within host cell cytoplasm

Carole Kebbi-Beghdadi, Ludovic Pilloux, Virginie Martin and Gilbert Greub*

Center for Research on Intracellular Bacteria, Institute of Microbiology, Centre Hospitalier Universitaire Vaudois, Lausanne, Switzerland

*Corresponding author:
Prof. Gilbert Greub
Institute of Microbiology
Rue du Bugnon 48
1011 Lausanne
Switzerland
Tel: 0041 21 314 4979
Fax 0041 21 314 4060
e-mail: gilbert.greub@chuv.ch

Supplementary Table 1:

Bacterial proteins retrieved in mass spectrometry analysis of infected eukaryotic cell cytosol.

HEp2 cells were infected with *C. trachomatis* or *W. chondrophila* and treated or not with perfringolysin O. The number of peptides allowing identification of a protein are indicated in PFO and mock columns. Molecular weight, presence of a predicted signal peptide and isoelectric point are from www.chlamdb.ch [35].

Identified Proteins	gene name	PFO	mock	fold change	Molecular weight	Signal peptide	Isoelectric point
**Chlamydia trachomatis**							
Chaperone protein DnaK OS=Chlamydia trachomatis (strain D/UW-3/Cx) GN=dnaK PE=3 SV=4	*dnaK*	18	0		71 kDa	NO	5.02
PqqC-like protein OS=Chlamydia trachomatis (strain D/UW-3/Cx) GN=CT_610 PE=1 SV=1	*CT_610*	11	0		27 kDa	NO	4.94
Elongation factor G OS=Chlamydia trachomatis (strain D/UW-3/Cx) GN=fusA PE=3 SV=1	*fusA*	8	0		77 kDa	NO	5.21
SWIB (YM74) complex protein OS=Chlamydia trachomatis (strain D/UW-3/Cx) GN=CT_460 PE=4 SV=1	*CT_460*	4	0		10 kDa	NO	9.7
Oligoendopeptidase OS=Chlamydia trachomatis (strain D/UW-3/Cx) GN=pepF PE=3 SV=1	*pepF*	4	0		69 kDa	NO	5.42
Thioredoxin OS=Chlamydia trachomatis (strain D/UW-3/Cx) GN=trxA PE=3 SV=1	*trxA*	4	0		11 kDa	NO	5.02
Elongation factor Ts OS=Chlamydia trachomatis (strain D/UW-3/Cx) GN=tsf PE=3 SV=1	*tsf*	3	0		31 kDa	NO	5.31
Hydrolase/phosphatase homolog OS=Chlamydia trachomatis (strain D/UW-3/Cx) GN=CT_771 PE=3 SV=1	*CT_771*	3	0		17 kDa	NO	5.04
Skp-like protein OS=Chlamydia trachomatis (strain D/UW-3/Cx) GN=CT_242 PE=3 SV=1	*CT_242*	3	0		19 kDa	YES	4.81
Trigger factor OS=Chlamydia trachomatis (strain D/UW-3/Cx) GN=tig PE=3 SV=1	*tig*	2	0		50 kDa	NO	5.01
Serine hydroxymethyltransferase OS=Chlamydia trachomatis (strain D/UW-3/Cx) GN=glyA PE=3 SV=1	*glyA*	2	0		54 kDa	NO	6.25
UPF0111 protein CT_691 OS=Chlamydia trachomatis (strain D/UW-3/Cx) GN=CT_691 PE=3 SV=1	*CT_691*	2	0		25 kDa	YES	4.95
Glyceraldehyde-3-phosphate dehydrogenase OS=Chlamydia trachomatis (strain D/UW-3/Cx) GN=gap PE=3 SV=1	*gap*	1	0		36 kDa	NO	5.58
50S ribosomal protein L7/L12 OS=Chlamydia trachomatis (strain D/UW-3/Cx) GN=rplL PE=3 SV=3	*rplL*	13	1	13	14 kDa	NO	4.9
Thio-specific Antioxidant (TSA) Peroxidase OS=Chlamydia trachomatis (strain D/UW-3/Cx) GN=ahpC PE=4 SV=1	*ahpC*	20	2	10	22 kDa	NO	4.77
Protein CT_858 OS=Chlamydia trachomatis (strain D/UW-3/Cx) GN=CT_858 PE=1 SV=2	*CT_858*	10	1	10	67 kDa	YES	5.68
Elongation factor Tu OS=Chlamydia trachomatis (strain D/UW-3/Cx) GN=tuf PE=3 SV=1	*tuf*	37	4	9.25	43 kDa	NO	5.36
DNA-directed RNA polymerase subunit alpha OS=Chlamydia trachomatis (strain D/UW-3/Cx) GN=rpoA PE=3 SV=1	*rpoA*	4	1	4	42 kDa	NO	5.34
60 kDa chaperonin OS=Chlamydia trachomatis (strain D/UW-3/Cx) GN=groL PE=1 SV=2	*groL*	61	25	2.44	58 kDa	NO	5.34
10 kDa chaperonin OS=Chlamydia trachomatis (strain D/UW-3/Cx) GN=groS PE=3 SV=1	*groS*	8	4	2	11 kDa	NO	4.99
30S ribosomal protein S6 OS=Chlamydia trachomatis (strain D/UW-3/Cx) GN=rpsF PE=3 SV=1	*rpsF*	3	2	1.5	13 kDa	NO	8.75
**Waddlia chondrophila**							
Uncharacterized protein wcw_0501 PE=4 SV=1	*wcw_0501*	5	0		47 kDa	YES	6.2
DO serine protease htrA3 wcw_0432 PE=4 SV=1	*htrA3*	4	0		52 kDa	YES	5.89
Uncharacterized protein wcw_0499 PE=4 SV=1	*wcw_0499*	3	0		18 kDa	NO	5.64
Nucleoside diphosphate kinase ndk wcw_1545 PE=3 SV=1	*ndk2*	3	0		18 kDa	YES	8.02
Putative (CPAF) cpa wcw_0991 PE=4 SV=1	*cpa*	3	0		67 kDa	YES	4.68
Putative Skp-like protein ompH wcw_1192 PE=4 SV=1	*ompH*	3	0		21 kDa	YES	5.52
Nucleoside diphosphate kinase ndk wcw_1543 PE=3 SV=1	*ndk1*	3	0		16 kDa	NO	5.22
Uncharacterized protein wcw_0657 PE=4 SV=1	*wcw_0657*	3	0		26 kDa	YES	5.12
Elongation factor Ts tsf wcw_1934 PE=3 SV=1	*tsf*	3	0		31 kDa	NO	5.26
50S ribosomal protein L7/L12 rplL wcw_0591 PE=3 SV=1	*rplL*	2	0		14 kDa	NO	4.93
Putative rhs family protein rhs11 wcw_0453 PE=4 SV=1	*rhs11*	2	0		200 kDa	YES	5.99
Uncharacterized protein wcw_0967 PE=4 SV=1	*wcw_0967*	2	0		31 kDa	YES	7.1
NADPH-dependent FMN reductase wcw_0878 PE=4 SV=1	*wcw_0878*	2	0		23 kDa	YES	8.59
Elongation factor G fusA wcw_0306 PE=3 SV=1	*fusA*	2	0		77 kDa	NO	5.09
60 kDa chaperonin groL wcw_1343 PE=3 SV=1	*groL*	1	0		58 kDa	NO	5.18
Peptidyl-prolyl cis-trans isomerase mip3 wcw_1529 PE=4 SV=1	*mip3*	1	0		28 kDa	YES	4.57
Uncharacterized protein wcw_0704 PE=4 SV=1	*wcw_0704*	1	0		526 kDa	NO	5.48
Uncharacterized protein wcw_1706 PE=4 SV=1	*wcw_1706*	1	0		28 kDa	NO	5.38
Chaperone protein DnaK dnaK wcw_1638 PE=2 SV=1	*dnaK*	1	0		70 kDa	NO	4.87
10 kDa chaperonin groES3 wcw_1848 PE=3 SV=1	*groES3*	1	0		11 kDa	NO	4.82
Translation initiation factor IF-1 infA wcw_0582 PE=3 SV=1	*infA*	1	0		8 kDa	NO	9.25
50S ribosomal protein L10 rplJ wcw_0590 PE=3 SV=1	*rplJ*	1	0		19 kDa	NO	5.14
Uncharacterized protein wcw_0680 PE=4 SV=1	*wcw_0680*	1	0		40 kDa	YES	5.13
RNA-binding protein rbp wcw_0715 PE=4 SV=1	*rbp*	1	0		10 kDa	NO	6.3
50S ribosomal protein L9 rplI wcw_0819 PE=3 SV=1	*rplI*	1	0		18 kDa	NO	5.36
Uncharacterized protein wcw_0969 PE=4 SV=1	*wcw_0969*	1	0		18 kDa	NO	4.81
Peptidyl-prolyl cis-trans isomerase ppiB wcw_1068 PE=3 SV=1	*ppiB*	1	0		22 kDa	YES	5.92
Uncharacterized protein wcw_1301 PE=4 SV=1	*wcw_1301*	1	0		17 kDa	YES	4.84
Elongation factor Tu tuf wcw_0584 PE=3 SV=1	*tuf*	7	1	7	43 kDa	NO	5.45
10 kDa chaperonin groS wcw_1342 PE=3 SV=1	*groS*	3	1	3	12 kDa	NO	5.85

Figures 1a and 1b

Wcw_0499

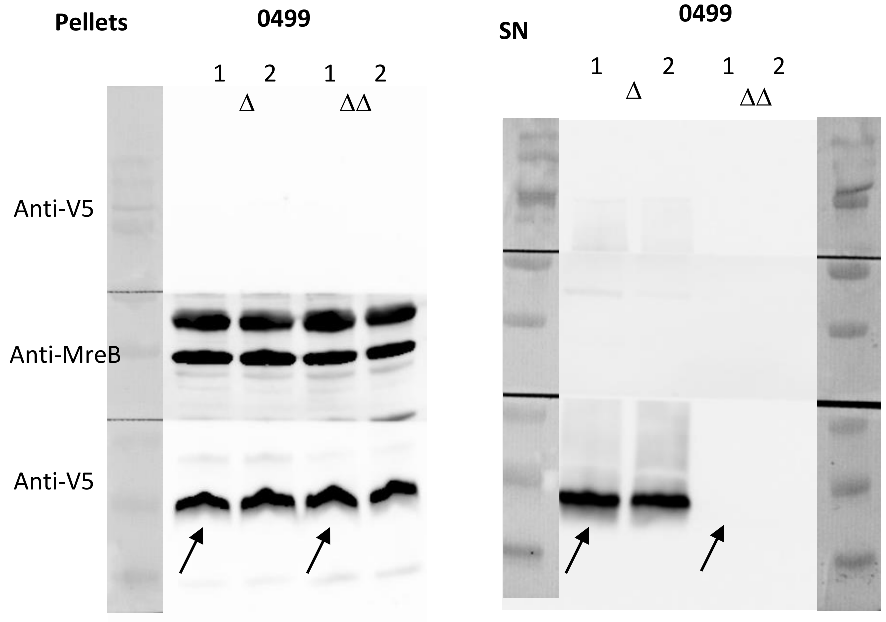

CT_432/CT_460

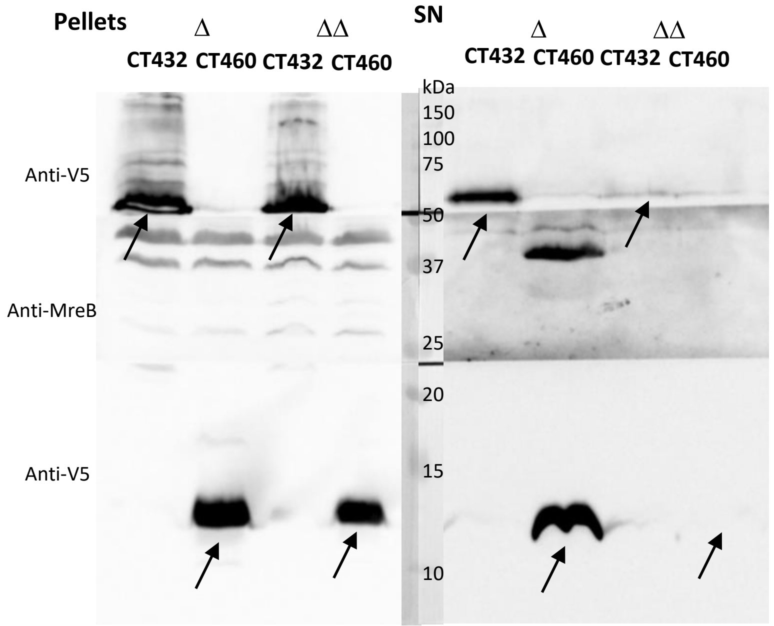

Wcw_1706

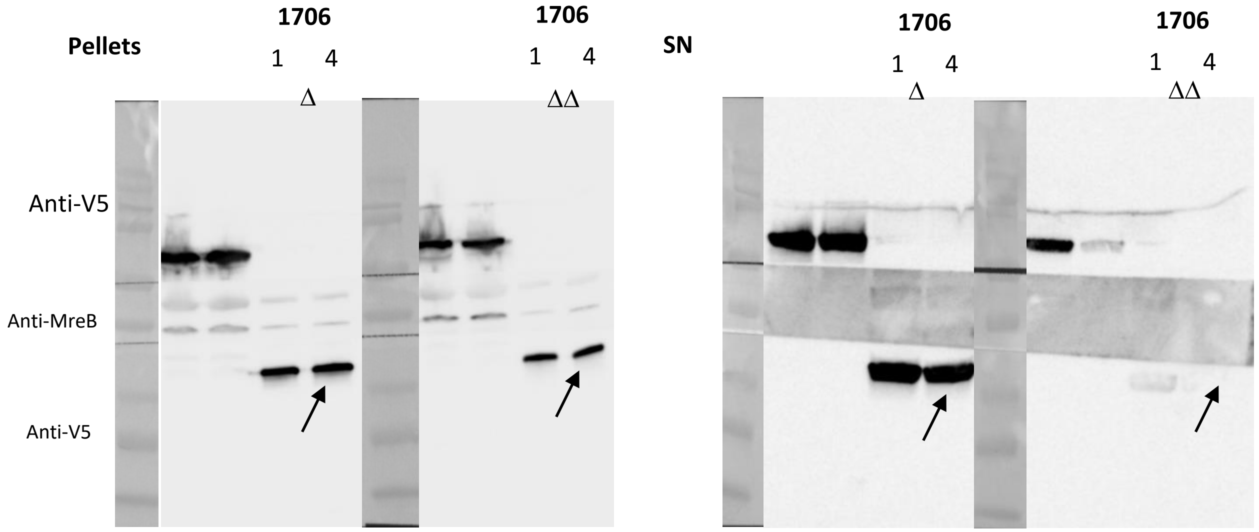

Supplementary Fig. S1: Full length immunoblots of secretion assays in *Y. enterocolitica* ΔHOPEMT and ΔHOPEMT ΔYscU strains. Epitope V5-tagged Wcw_0499, Wcw_1706, CT432 and CT460 were detected in the bacterial pellet or in the culture supernatant (SN). Experiments were performed in duplicates. Arrows indicate bands that are presented in Figures 1a and 1b.

Figure 1c: controls

CPAF

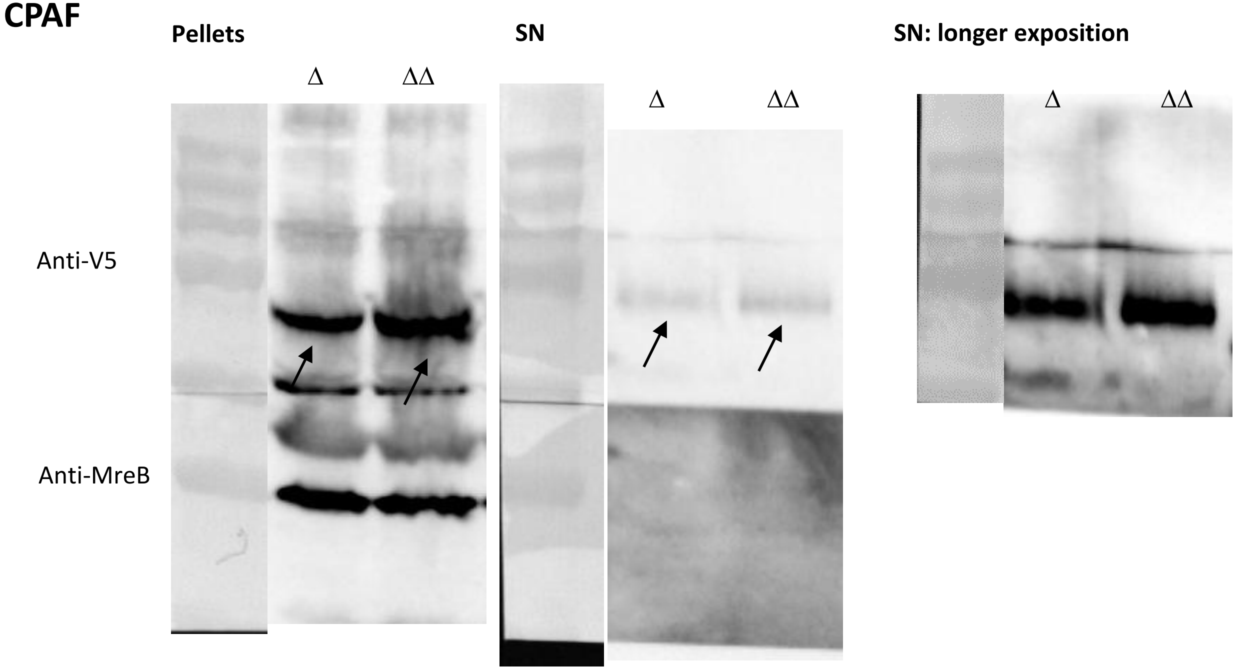

TepP

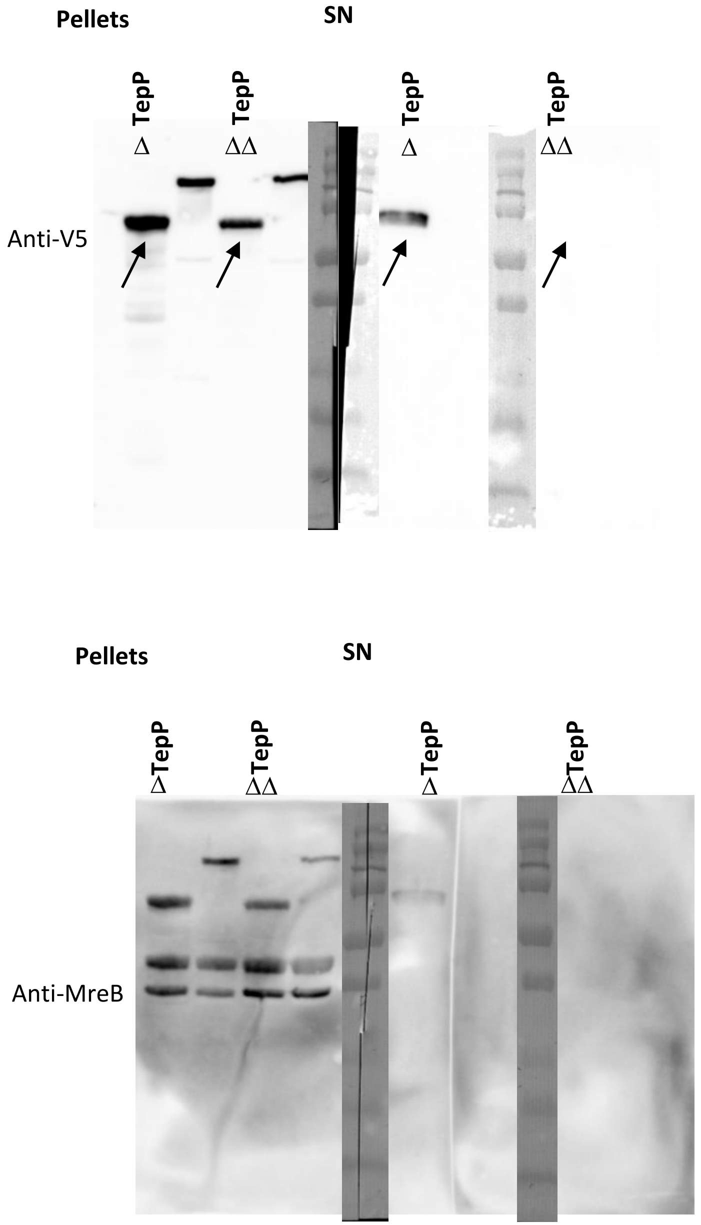

Supplementary Fig. S2: Full length immunoblots of secretion assays in *Y. enterocolitica* ΔHOPEMT and ΔHOPEMT ΔYscU strains. *W. chondrophila* CPAF (Wcw_0991) and *C. trachomatis* TepP *(*CT875) were tagged with a V5 epitope and detected by western blot in the bacterial pellet or in the culture supernatant (SN). The absence of the strictly cytosolic *Y. enterocolitica* MreB protein in SN fractions confirms that there was no bacterial lysis. Arrows indicate bands that are presented in Figure 1c.

Figure 1c: controls

TEM without SP

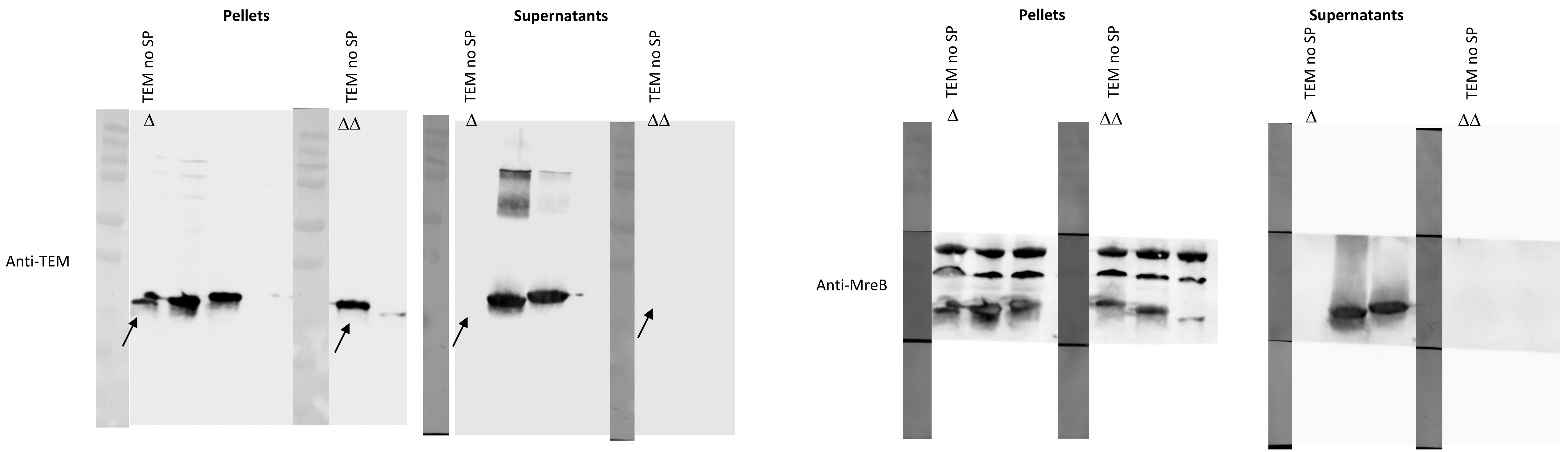

Supplementary Fig. S3: Full length immunoblots of secretion assays in *Y. enterocolitica* ΔHOPEMT and ΔHOPEMT ΔYscU strains. A truncated form (no signal peptide) of the *E. coli* β lactamase (TEM without SP) was detected by western blot in the bacterial pellet or in the culture supernatant (SN). The absence of the strictly cytosolic *Y. enterocolitica* MreB protein in SN fractions confirms that there was no bacterial lysis. Arrows indicate bands that are presented in Figure 1c.

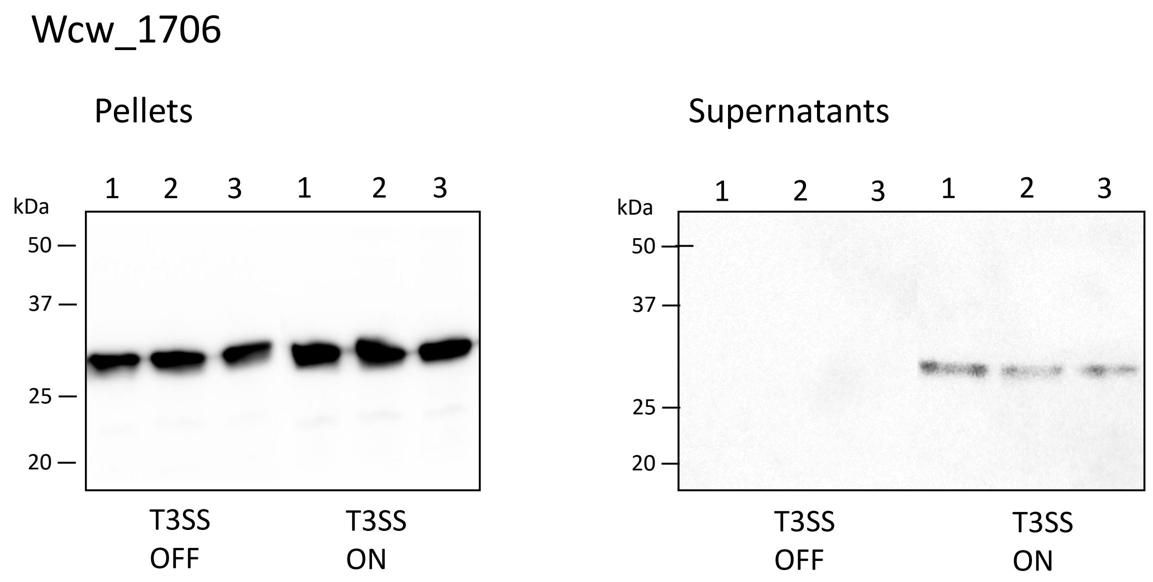

Supplementary Fig. S4: Wcw_1706 was detected by immunoblot in the bacterial pellet or in the culture supernatant of *Y. enterocolitica* grown in presence (T3SS OFF) or in absence (T3SS ON) of calcium following the procedure described in [33].

Figure 4b

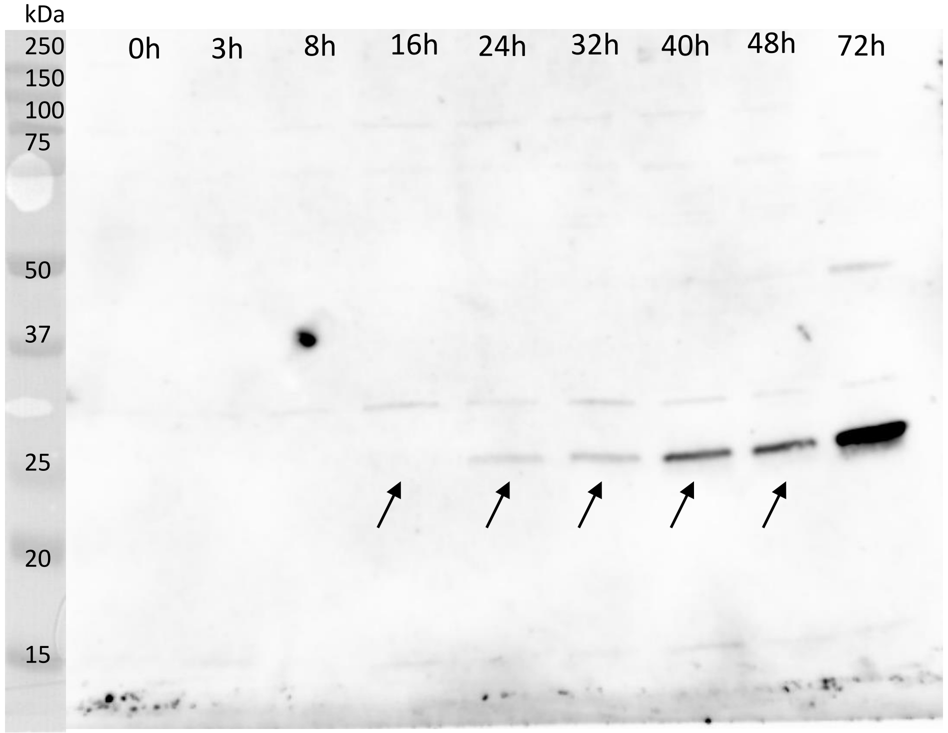

Supplementary Fig. S5: Full length immunoblot of Wcw_1706 protein expression at different time points during the course of *W. chondrophila* infection in Vero cells. Arrows indicate bands that are presented in Figure 4b.

Figure 4d

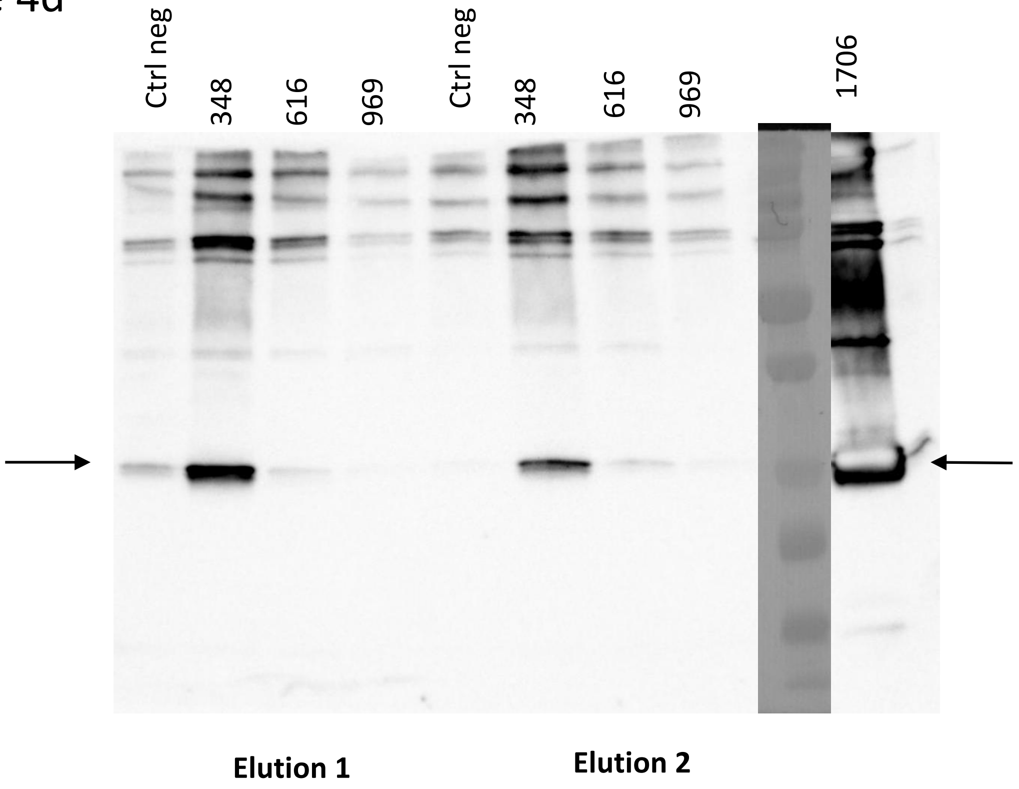

Supplementary Fig. S6: Full length immunoblot of pull down experiments with Wcw_1706 and T3SS chaperones Wcw_0969, Wcw_0616 and Wcw_0348. Negative control = no protein. A mouse polyclonal anti-Wcw_1706 antibody was used to probe the blot. Arrows indicate bands that are presented in Figure 4d.

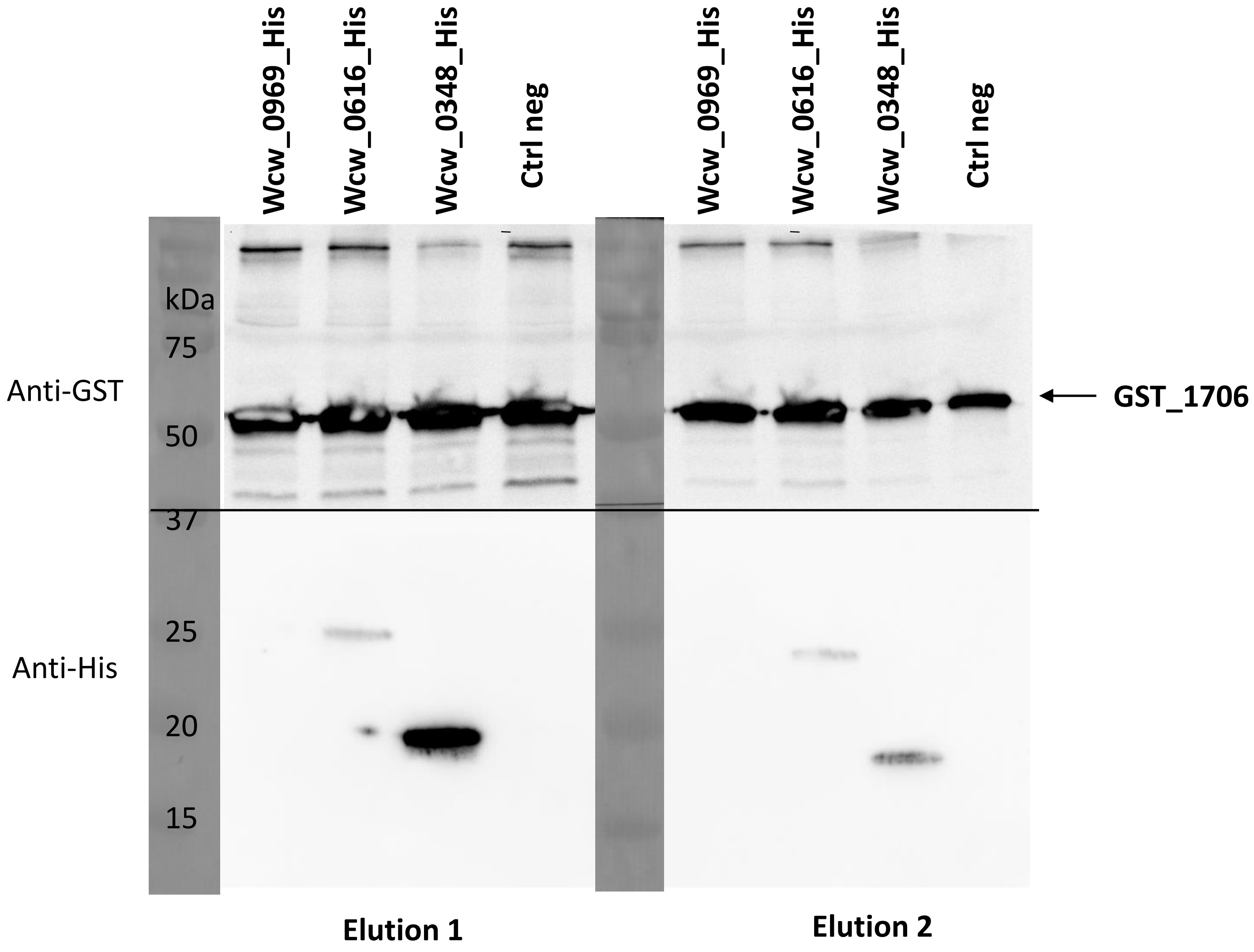

Supplementary Fig. S7: Immunoblot of a GST-pull down experiment involving a GST-tagged version of Wcw_1706 and His-tagged T3SS chaperones Wcw_0969, Wcw_0616 and Wcw_0348. Negative control = no protein. Mouse monoclonal anti-GST and anti-His antibodies were used to probe the blot.
